# Peroxisome proliferator‐activated receptor γ coactivator 1α regulates mitochondrial calcium homeostasis, sarcoplasmic reticulum stress, and cell death to mitigate skeletal muscle aging

**DOI:** 10.1111/acel.12993

**Published:** 2019-07-10

**Authors:** Jonathan F. Gill, Julien Delezie, Gesa Santos, Shawn McGuirk, Svenia Schnyder, Stephan Frank, Martin Rausch, Julie St‐Pierre, Christoph Handschin

**Affiliations:** ^1^ Biozentrum, Division of Pharmacology/Neurobiology University of Basel Basel Switzerland; ^2^ Department of Biochemistry, Rosalind and Morris Goodman Cancer Centre McGill University Montreal Quebec Canada; ^3^ Division of Neuropathology, Institute of Pathology, University Hospital Basel University of Basel Basel Switzerland; ^4^ Biotherapeutic and Analytical Technologies Novartis Institutes for BioMedical Research (NIBR) Basel Switzerland; ^5^ Present address: Department of Biochemistry, Microbiology, and Immunology University of Ottawa Ottawa Ontario Canada

**Keywords:** aging, calcium homeostasis, cell death, health span, mitochondria, PGC‐1α, skeletal muscle, tubular aggregates

## Abstract

Age‐related impairment of muscle function severely affects the health of an increasing elderly population. While causality and the underlying mechanisms remain poorly understood, exercise is an efficient intervention to blunt these aging effects. We thus investigated the role of the peroxisome proliferator‐activated receptor γ coactivator 1α (PGC‐1α), a potent regulator of mitochondrial function and exercise adaptation, in skeletal muscle during aging. We demonstrate that PGC‐1α overexpression improves mitochondrial dynamics and calcium buffering in an estrogen‐related receptor α‐dependent manner. Moreover, we show that sarcoplasmic reticulum stress is attenuated by PGC‐1α. As a result, PGC‐1α prevents tubular aggregate formation and cell death pathway activation in old muscle. Similarly, the pro‐apoptotic effects of ceramide and thapsigargin were blunted by PGC‐1α in muscle cells. Accordingly, mice with muscle‐specific gain‐of‐function and loss‐of‐function of PGC‐1α exhibit a delayed and premature aging phenotype, respectively. Together, our data reveal a key protective effect of PGC‐1α on muscle function and overall health span in aging.

## 1 INTRODUCTION

Muscle strength and mass progressively decline with age, leading to physical disability, and ultimately higher morbidity and mortality. Although the cause of muscle aging is multifactorial, reduced mitochondrial function is a commonly observed phenomenon associated with muscle deterioration during aging (Peterson, Johannsen, & Ravussin, 2012). For example, reduced mitochondrial biogenesis, decreased mitochondrial mass and aberrant fission to fusion rates concomitant with a mitochondrial turnover drop have been reported in old muscle. In addition, increased mitochondrial DNA damage, depolarized and swollen mitochondria, diminished rates of oxidative phosphorylation (OXPHOS) and Krebs cycle activity have been found in this context. Collectively, the ensuing reduced ATP production, impaired calcium homeostasis, elevated apoptosis, and increased levels of reactive oxygen species (ROS) all contribute to age‐associated muscle dysfunction.

Importantly, mitochondria, together with the sarcoplasmic reticulum (SR), control cellular calcium homeostasis (Rizzuto, Stefani, Raffaello, & Mammucari, 2012). These two organelles communicate via contact sites, termed mitochondria‐associated ER membranes (MAMs), focal hotspots for the exchange of calcium as well as the synthesis and transfer of phospholipids, initiation of mitochondrial fission, mitophagy, and signal transduction events (Marchi, Patergnani, & Pinton, 2014). Aging impairs mitochondria‐SR association and mitochondrial calcium uptake (Pietrangelo et al., 2015), which can contribute to a dysregulation of cellular calcium homeostasis. Furthermore, all three structures, mitochondria, MAMs, and the SR, have been linked to the initiation of cell death (Danese et al., 2017) and increased apoptosis levels in old muscle (Whitman, Wacker, Richmond, & Godard, 2005). For example, apoptosis can be mediated in mitochondria via caspase‐dependent and caspase‐independent pathways, both of which are rising with age (Siu, Pistilli, & Alway, 2005). In addition, mitochondrial and nuclear DNA damage due to increased ROS levels also contributes to age‐related muscle apoptosis (Harman, 2006). Finally, endoplasmic reticulum (ER) stress is also a strong promoter of apoptotic events (Szegezdi, Logue, Gorman, & Samali, 2006).

Important regulators of mitochondrial biogenesis and function, most notably the peroxisome proliferator‐activated receptor γ coactivator 1α (PGC‐1α), are reduced in skeletal muscle in the aging process and have been associated with the decrease in mitochondrial function (Ling et al., 2004). Importantly, this deterioration can partly be restored by exercise (Kang, Chung, Diffee, & Ji, 2013), a strong promoter of PGC‐1α transcription and activity. In addition, transgenic overexpression of PGC‐1α extends lifespan in *Drosophila melanogaster* (Rera et al., 2011). Using gain‐of‐function and loss‐of‐function mouse models for muscle PGC‐1α, we aimed at providing a unifying and comprehensive study determining the role of PGC‐1α in muscle aging, in particular in regard to calcium homeostasis and cell death.

## 2 RESULTS

### 2.1 PGC‐1α modulates mitochondrial dynamics and SR association in the aging muscle

**Figure 1:**
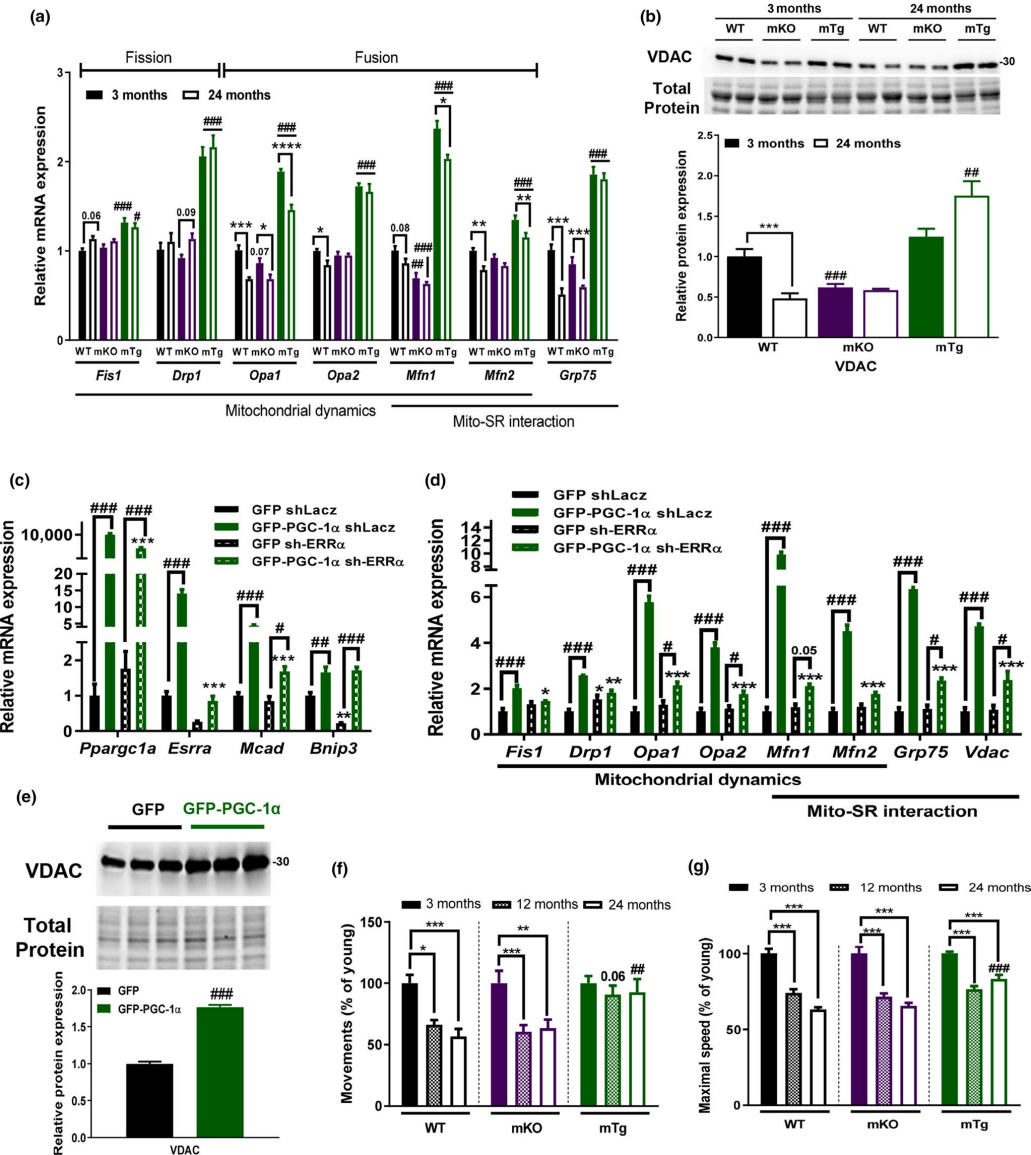
Peroxisome proliferator‐activated receptor γ coactivator 1α (PGC‐1α) increases mitochondrial dynamics and sarcoplasmic reticulum (SR) association in an estrogen‐related receptor α (ERRα)‐dependent manner and improves muscle function during aging. (a) Relative muscle mRNA levels of genes related to mitochondrial dynamics and SR association (*n* = 6). (b) Relative muscle voltage‐dependent anion channel (VDAC) protein levels (*n* = 6). (c and d) Relative C2C12 cell mRNA levels of PGC‐1α and ERRα targets and of genes related to mitochondrial dynamics and SR association (*n* = 3 independent experiments with three technical replicates). (e) Relative C2C12 cell VDAC protein levels (*n* = 3 independent experiments with three technical replicates). (f) Age‐related reduction in spontaneous locomotor activity measured by the CLAMS system. Values represent 48 hr of recording (*n* = 8–9). (g) Age‐related reduction in maximal running speed during treadmill exhaustion test (*n* = 10–12). Values are mean ± *SEM*. *p < 0.05; **p < 0.01; ***p < 0.001 indicate statistically significant differences between young and old animals of the same genotype or between cells with endogenous and overexpressed ERRα levels, ^#^ *p* < 0.05; ^##^ *p* < 0.01; ^###^ *p* < 0.001 indicate statistically significant differences between genotypes of age‐matched animals or between cells with endogenous and overexpressed PGC‐1α levels

In light of the strong decline in mitochondrial function in aging, we first performed a broad comparative analysis of young and old wild‐type (WT), PGC‐1α muscle‐specific transgenic (mTg‐PGC‐1α), and PGC‐1α muscle‐specific knockout (mKO‐PGC‐1α) mice. In these skeletal muscle‐specific gain‐of‐function and loss‐of‐function models for PGC‐1α, many of these parameters have been extensively assessed and reported in young animals (Handschin, Chin, et al., 2007; Handschin, Choi, et al., 2007; Lin et al., 2002), but data in old mice are scarce. Mitochondrial fission and fusion events are essential for proper mitochondrial physiology, but these programs controlling mitochondrial dynamics decline in aging (Seo et al., 2010). In skeletal muscle of 24‐month‐old WT mice, we observed, along with a severe reduction of the *Pgc‐1α* transcript (Figure S1a), an age‐dependent decreased expression of the fusion genes *Mitofusin 1 and 2* (*Mfn1* and *2*) and *Optic atrophy 1 and 2* (*Opa1* and *2*; Figure 1a). Furthermore, muscle OXPHOS protein content was diminished in 24‐month‐old WT mice (Figure S1b). Intriguingly, loss of PGC‐1α in muscles of young mKO‐PGC‐1α animals decreased OXPHOS protein (Figure S1b) and *Mfn1* gene expression levels (Figure 1a) to values comparable to those obtained from aged WT muscles. Conversely, PGC‐1α overexpression in young mTg‐PGC‐1α muscles strikingly upregulated the levels of *Mfn1‐2* and *Opa1‐2*, as well as OXPHOS proteins, and prevented the age‐linked reduction of these genes and proteins (Figure 1a; Figure S1b). In addition, muscle PGC‐1α overexpression increased mtDNA amount as well as both mitochondrial density and size (Figure S2a–c), correlating with elevated expression of the estrogen‐related receptor α (*Esrra*, ERRα) and the mitochondrial transcription factor A (*Tfam),* essential regulators of mitochondrial biogenesis and function (Figure S2d).

Besides their role in mitochondrial dynamics, mitofusin proteins, in particular *Mfn2*, constitute essential elements of MAMs, linking the SR and the mitochondrial compartments. In parallel with diminished *Mfn1* and *2* expression upon aging in WT muscles, a significant reduction of other essential mediators of the mitochondria‐SR communication, such as the glucose‐regulated protein 75 (GRP75) and the voltage‐dependent anion channel (VDAC), was detected (Figure1a,b). Interestingly, loss of PGC‐1α in young mKO‐PGC‐1α muscles also decreased VDAC protein expression, while PGC‐1α overexpression in mTg‐PGC‐1α mice and C2C12 cells resulted in higher levels of GRP75 gene and VDAC protein expression, independent of age of the animal (Figure 1a–e). We further demonstrated that overexpression of PGC‐1α in C2C12 cells induces the expression of *Mfn1* and *2*, *Drp1*, *Fission 1* (*Fis1*), *Opa1*‐*2*, *Grp75,* and VDAC in an ERRα‐dependent manner (Figure 1d,e). Other PGC‐1α target genes, for example, the BCL2/adenovirus E1B 19‐kDa interacting protein 3 (*Bnip3*), were not affected by ERRα knockdown, (Figure 1c). The blunting effects of PGC‐1α overexpression on age‐induced mitochondrial changes were associated with a mitigation of the age‐related reduction in spontaneous locomotion and endurance performance (Figure 1f,g and Figure S1c,d). In contrast, muscle‐specific PGC‐1α deletion led to a premature decrease in running capacity in 12‐month‐old mKO animals (Figure S1c). These data correlate with the balance, muscle strength, and fiber size changes upon PGC‐1α modulation in old animals (Gill, Santos, Schnyder, & Handschin, 2018). Together, these results clearly show that PGC‐1α is not only an essential regulator of mitochondrial function and dynamics in young and old animals, but also prevents the age‐associated decline of these systems.

### 2.2 PGC‐1α improves mitochondrial calcium handling during aging

**Figure 2:**
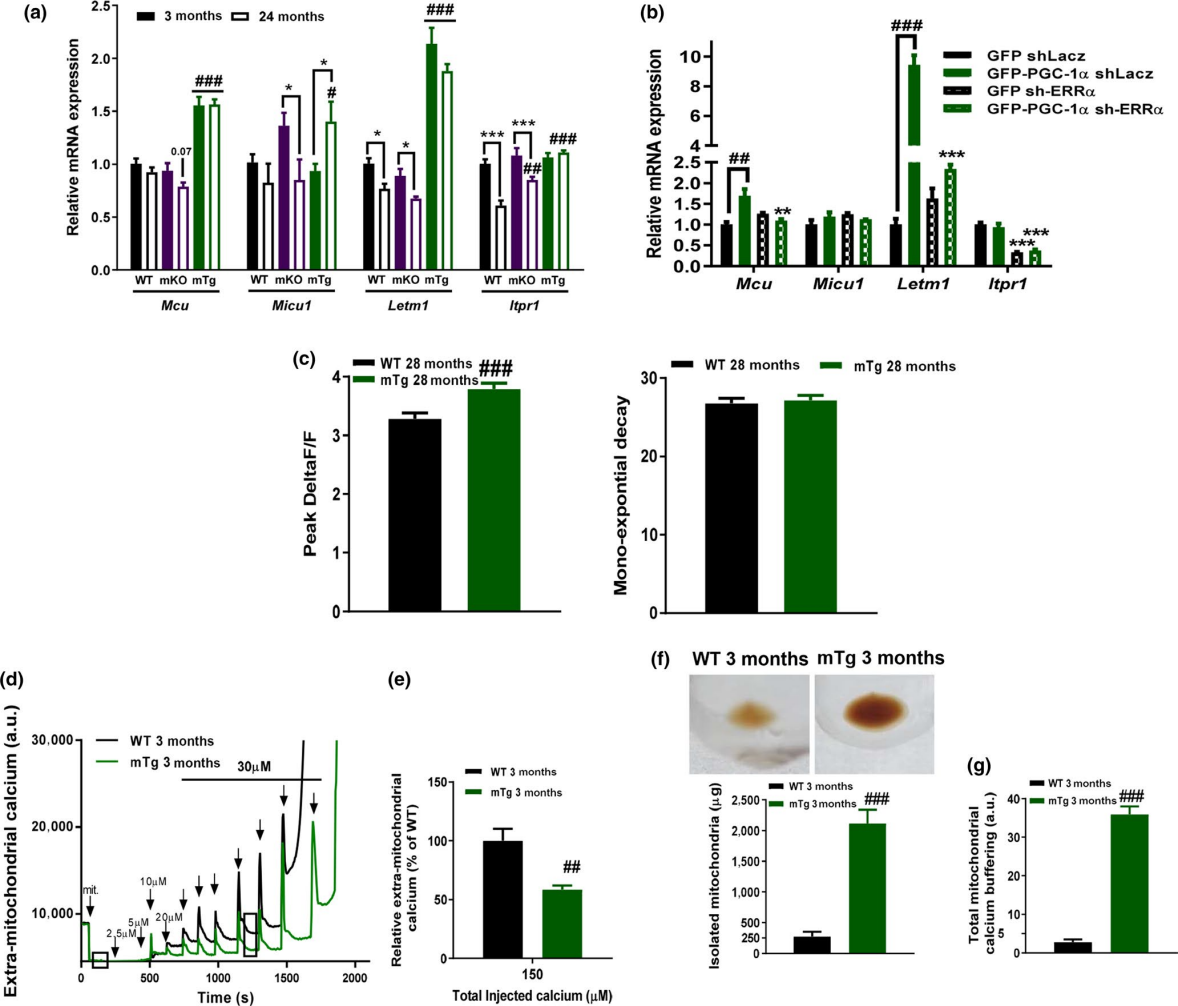
Peroxisome proliferator‐activated receptor γ coactivator 1α (PGC‐1α) ameliorates mitochondrial calcium buffering. (a) Relative muscle mRNA levels of mitochondrial calcium buffering genes (*n* = 6). (b) Relative C2C12 myoblasts mRNA levels of mitochondrial calcium buffering genes (*n* = 3 independent experiments with three technical replicates). (c) Relative mitochondrial calcium uptake (left panel) and removal (right panel) in FDB fibers of 28‐month‐old animals. (d and e) Representative extra‐mitochondrial calcium traces upon calcium injection and quantification of relative extra‐mitochondrial calcium levels after a total addition of 150 μm of calcium. Squares indicate the baseline and assay values used for quantifications shown in (e) (*n* = 4; two independent experiments with two muscles of each genotype used in each experiment). (f) Representative pictures of mitochondrial pellets after mitochondria isolation from hindlimb muscles and measure of isolated mitochondrial quantity. (g) Total mitochondrial calcium buffering capacity calculated by multiplying total mitochondrial quantity by the percentage of calcium imported in the mitochondria. Values are mean ± *SEM*. *p < 0.05; **p < 0.01; ***p < 0.001 indicate statistically significant differences between young and old animals of the same genotype or between cells with endogenous and overexpressed estrogen‐related receptor α (ERRα) levels, ^#^ *p* < 0.05; ^##^ *p* < 0.01; ^###^ *p* < 0.001 indicate statistically significant differences between genotypes of age‐matched animals or between cells with endogenous and overexpressed PGC‐1α levels

A disrupted mitochondrial network, specifically the mitochondria‐SR association, affects cellular calcium homeostasis (Fernandez‐Sanz et al., 2014; Pietrangelo et al., 2015). Our group has previously demonstrated that PGC‐1α modulates SR‐controlled calcium levels in skeletal muscle (Summermatter et al., 2012), but the involvement of PGC‐1α in regulating mitochondrial calcium uptake capacity and the exchange between mitochondria and the SR is unknown. We observed that aging significantly reduced the expression of genes involved in mitochondrial calcium uptake and transfer from the SR, including the leucine zipper and EF‐hand containing transmembrane protein 1 (*Letm1*) and the inositol 1,4,5‐trisphosphate (IP3) receptor type 1 (*Itpr1*) in WT muscles (Figure 2a). Interestingly, PGC‐1α upregulation prevented the age decline of *Iptr1* and *Letm1* and significantly upregulated genes related to mitochondrial calcium buffering in the muscle of old mice, including the mitochondrial calcium uniporter (*Mcu*) gene (Figure 2a). The effect of PGC‐1α on *Mcu* and *Letm1* was recapitulated in cultured myotubes (Figure 2b). The PGC‐1α‐mediated induction of the expression of these two genes was furthermore dependent on ERRα (Figure 2b). Intriguingly, the genes encoding proteins related to calcium exchange that form an organelle‐spanning complex to control mitochondria‐SR interactions at MAMs were all downregulated in old WT mice, and rescued in old mTg‐PGC‐1α animals, including *Itpr1* on the SR side, *Grp75* providing a link between SR and mitochondria, and *Vdac* on the mitochondrial side. The tethering of this complex to both organelles is supported by the additional link provided by the mitofusins, which exhibit a similar regulation. Of note, this age‐related dysregulation was at least in part exacerbated by knockout of PGC‐1α. To test whether these changes affect calcium handling, mitochondrial calcium uptake in flexor digitorum brevis (FDB) muscle fibers isolated from old mTg‐PGC‐1α and WT mice was assessed. In line with the increased expression of MAM calcium exchange genes, mitochondria in FDB fibers of old muscle buffered more calcium when comparing mTG‐PGC‐1α to WT mice (Figure 2c). Calcium removal kinetics were however not different between the genotypes (Figure 2c). Next, the autonomous calcium buffering capacity of isolated mitochondria was investigated. We found that after the addition of 150 µM of calcium, calcium uptake was higher in mTg‐PGC‐1α compared to WT mitochondria (Figure 2d,e and Figure S2e). Since equal amounts of isolated mitochondria (275 µg) were used for these measurements, the results indicate that when normalized to mitochondrial amount, mitochondria from mTg‐PGC‐1α animals have an improved intrinsic calcium buffering capacity compared to mitochondria from WT mice. Thus, when considering the higher total mitochondrial mass in the gain‐of‐function model (Figure 2f), the total mitochondrial calcium buffering capacity of skeletal muscle in mTg‐PGC‐1α animals is even further amplified (Figure 2g).

### 2.3 PGC‐1α prevents ER stress and tubular aggregate formation in the aging muscle

**Figure 3:**
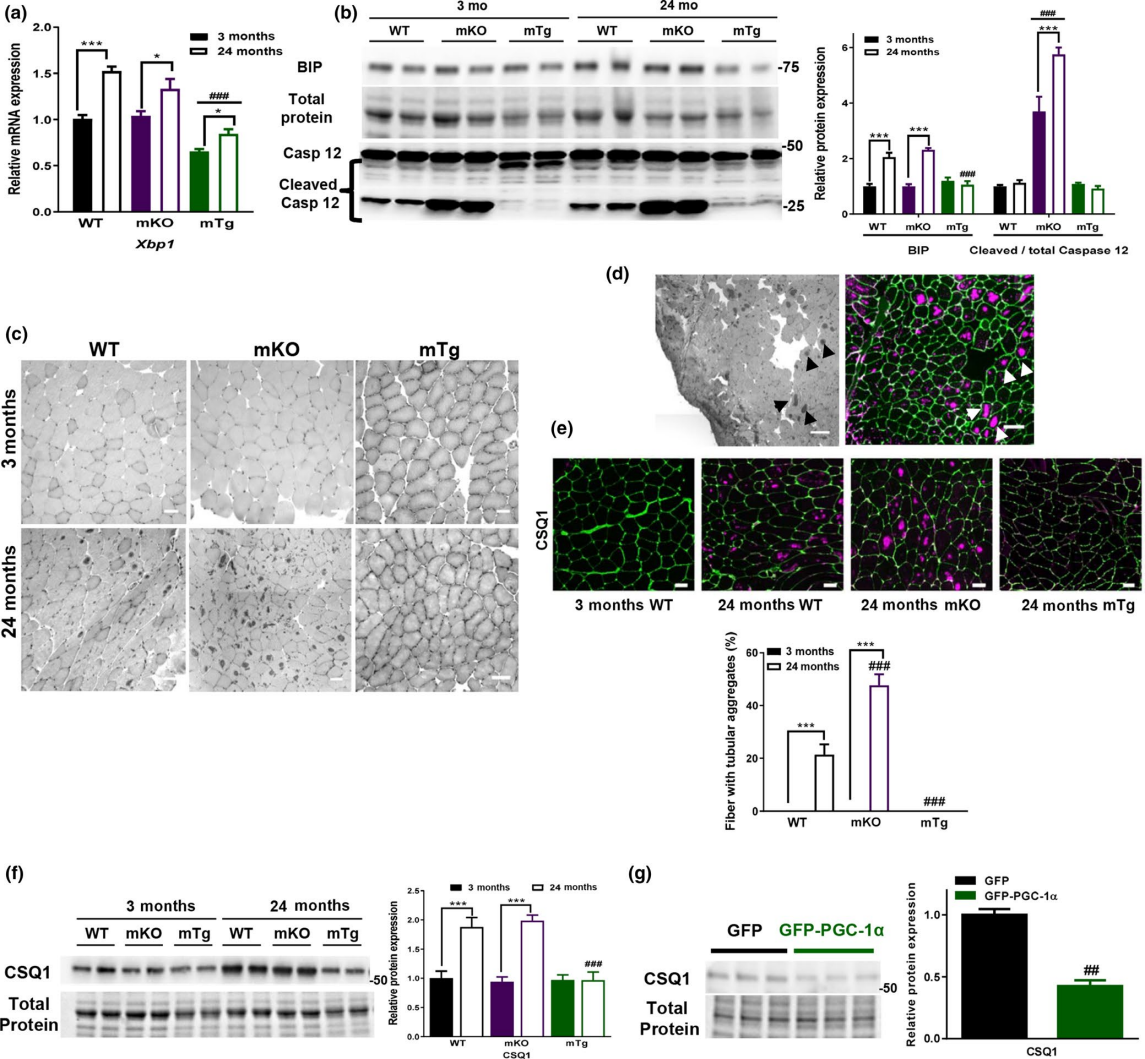
Peroxisome proliferator‐activated receptor γ coactivator 1α (PGC‐1α) reduces endoplasmic reticulum (ER) stress during aging and prevents age‐related tubular aggregate formation. (a and b) Relative muscle mRNA and protein levels of ER stress‐related genes (*n* = 5–6). (c) Representative pictures of H&E‐stained tibialis anterior cryosection, scale bars represent 50 µm. (d) Colocalization of H&E labeled aggregates (left picture) with calsequestrin 1 staining (right picture) indicated by arrows, scale bars represent 100 µm. (e) Representative pictures of tubular aggregates stained with calsequestrin 1 antibody and quantification of the percentage of fibers containing tubular aggregates, scale bars represent 50 µm (*n* = 6). (f and g) Calsequestrin 1 protein levels in muscles of young and old animals and in C2C12 myotubes (*n* = 3 independent experiments with three technical replicates). Values are mean ± *SEM*. *p < 0.05; **p < 0.01; ***p < 0.001 indicate statistically significant differences between young and old animals of the same genotype, ^#^ *p* < 0.05; ^##^ *p* < 0.01; ^###^ *p* < 0.001 indicate statistically significant differences between genotypes of age‐matched animals or between cells with endogenous and overexpressed PGC‐1α levels

Dysregulated calcium exchange between the ER and mitochondria is linked to ER stress (Malhotra & Kaufman, 2011). To test whether the modulation in mitochondria‐SR interaction and mitochondrial calcium buffering affects the SR, the extent of ER stress was investigated in the muscles of our mouse cohorts. We observed that ER stress increases with age as demonstrated by elevated expression of the X‐box binding protein 1 (Xbp1) and the chaperone protein BIP in WT and mKO‐PGC‐1α muscles, but not to the same extent in mTg‐PGC‐1α animals (Figure 3a,b). Furthermore, PGC‐1α muscle deletion led to an increased activation of the calcium stress marker caspase 12, which was exacerbated with age (Figure 3b). Consistently, poly‐ubiquitination of proteins, indicative of proteasomal degradation of potentially misfolded proteins, was dramatically increased in mKO‐PGC‐1α muscles with age and reduced in both young and old muscles of mTg‐PGC‐1α mice (Figure S3a). Of note, the in vivo reduction of *Xbp1* mRNA levels and protein poly‐ubiquitination by PGC‐1α were recapitulated in differentiated C2C12 cells (Figure S3b,c).

Overload of SR function results in a compensatory increase in SR membranes and ultimately the development of tubular aggregates, for example, as reported in muscle fibers of old mice (Agbulut, Destombes, Thiesson, & Butler‐Browne, 2000). On histological sections, we noticed the presence of abnormal eosin‐labeled structures in tibialis anterior (TA) muscles of old WT and mKO‐PGC‐1α animals (Figure 3c), confirmed as tubular aggregates by positive staining for the SR marker calsequestrin 1 (CSQ1) in TA (Figure 3d), and electron microscopy‐based analysis of old WT and mKO‐PGC‐1α gastrocnemius muscles (Figure S4a). Interestingly, the age‐dependent occurrence of tubular aggregates doubled in TA muscles of old mKO‐PGC‐1α compared age‐matched WT animals (Figure 3e). Remarkably, no tubular aggregates were detected in fast and mixed muscles of aged mTg‐PGC‐1α mice (Figure 3c,e and Figure S4a). Tubular aggregates were likewise absent in the old oxidative soleus muscle (Figure S4b) and reduced succinate dehydrogenase (SDH)‐positive fibers of other muscle beds in old WT and mKO‐PGC‐1α mice (Figure S4c), suggesting a link to the respective metabolic fiber type. The formation of tubular aggregates is closely associated with the accumulation of CSQ1 and other SR proteins, which might subsequently overwhelm the unfolded protein response (Chevessier et al., 2005). Thus, along with the absence of tubular aggregate formation in muscle of old mTg‐PGC‐1α mice, elevation of muscle PGC‐1α levels prevented the age‐related increase of CSQ1 protein levels that is observed in WT and mKO‐PGC‐1α mice (Figure 3f). The acute reduction of CSQ1 protein and transcript levels in cultured myotubes overexpressing PGC‐1α implies that this effect is at least in part directly linked to PGC‐1α, and not only relies on indirect, secondary effects of a fiber‐type shift (Figure 3g). Of note, in addition to the tubular aggregates, electron microscopy also revealed other age‐linked, abnormal structures of unknown origin and function in muscles fibers of old mKO‐PGC‐1α and WT mice that were absent from the young animals of all three genotypes and from old mTg‐PGC‐1α mice (Figure S4d), indicating further age‐associated abnormalities and damage in skeletal muscle that can be prevented by high levels of PGC‐1α.

### 2.4 PGC‐1α reduces the activation of cell death pathways in muscle of old animals

**Figure 4:**
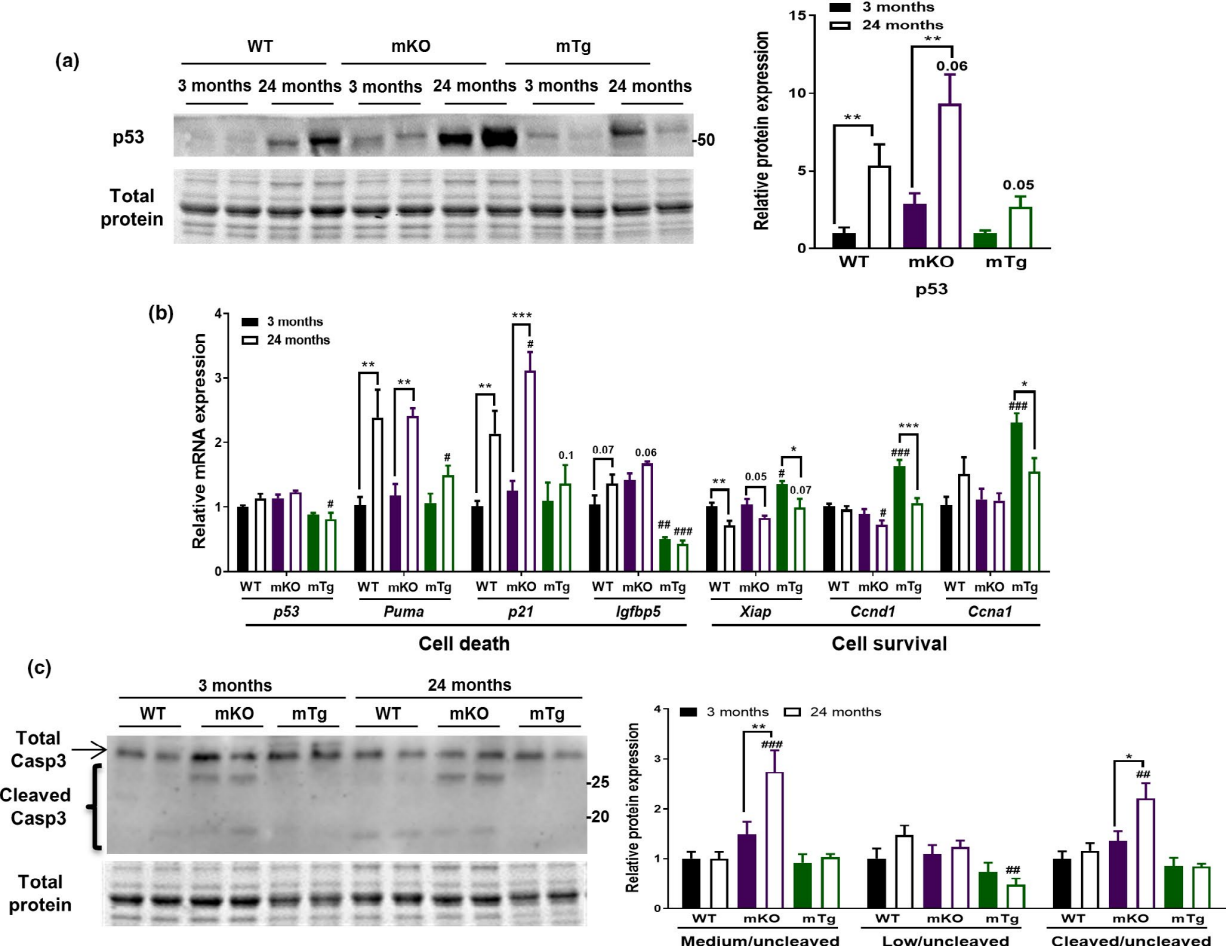
Peroxisome proliferator‐activated receptor γ coactivator 1α (PGC‐1α) inhibits age‐related muscle cell death. (a–c) Relative muscle mRNA and protein levels of cell death and cell survival‐related genes (*n* = 5–6). Values are mean ± *SEM*. *p < 0.05; **p < 0.01; ***p < 0.001 indicate statistically significant differences between young and old animals of the same genotype, ^#^ *p* < 0.05; ^##^ *p* < 0.01; ^###^ *p* < 0.001 indicate statistically significant differences between genotypes of age‐matched animals

Abnormal mitochondrial function, ER stress response, mitochondria‐SR interaction, and calcium homeostasis have all been described in the context of the initiation of cell death (Danese et al., 2017). We therefore assessed whether age‐associated apoptosis in skeletal muscle was affected by modulation of PGC‐1α. P53, a major regulator of cell death induction upon DNA damage and other stress conditions, was upregulated during aging of WT and mKO‐PGC‐1α muscles, but not in mTg‐PGC‐1α muscle overexpressing PGC‐1α (Figure 4a). Compared to aged WT animals, muscles of old mKO‐PGC‐1α and mTg‐PGC‐1α mice exhibited, respectively, trends toward a twofold increase (*p* = 0.06) and a twofold decrease (*p* = 0.05) in P53 protein levels. Interestingly, mRNA levels of the pro‐apoptotic insulin‐like growth factor binding protein 5 (*Igfbp5*) and of the p53 targets *p21* and BCL2‐binding component 3 (*Puma)* followed a similar pattern of expression, implying increased activity of P53 in old WT and mKO‐PGC‐1α, but not mTg‐PGC‐1α mice (Figure 4b). The transcript levels of the cell survival‐related gene X‐linked inhibitor of apoptosis protein (*Xiap*) were downregulated with age in WT and mKO‐PGC‐1α muscles and cyclin D transcript (*Ccnd1*) expression was lower in old mKO‐PGC‐1α muscles relative to age‐matched WT muscles. PGC‐1α significantly elevated the expression of all pro‐survival genes in young, and *Xiap* mRNA levels in old, muscles of mTg‐PGC‐1α mice (Figure 4b). Caspase 3 cleavage, a marker of cell death, was increased with age in mKO‐PGC‐1α mice and significantly higher in muscles of old mKO‐PGC‐1α mice relative to muscles of age‐matched WT animals (Figure 4c). Additionally, in old muscle tissues, the smallest cleavage product of caspase 3 was reduced by PGC‐1α overexpression. Taken together, our findings suggest a protective function of muscle PGC‐1α against age‐induced muscle cell death.

### 2.5 PGC‐1α protects against ceramide‐ and thapsigargin‐induced cell death

**Figure 5:**
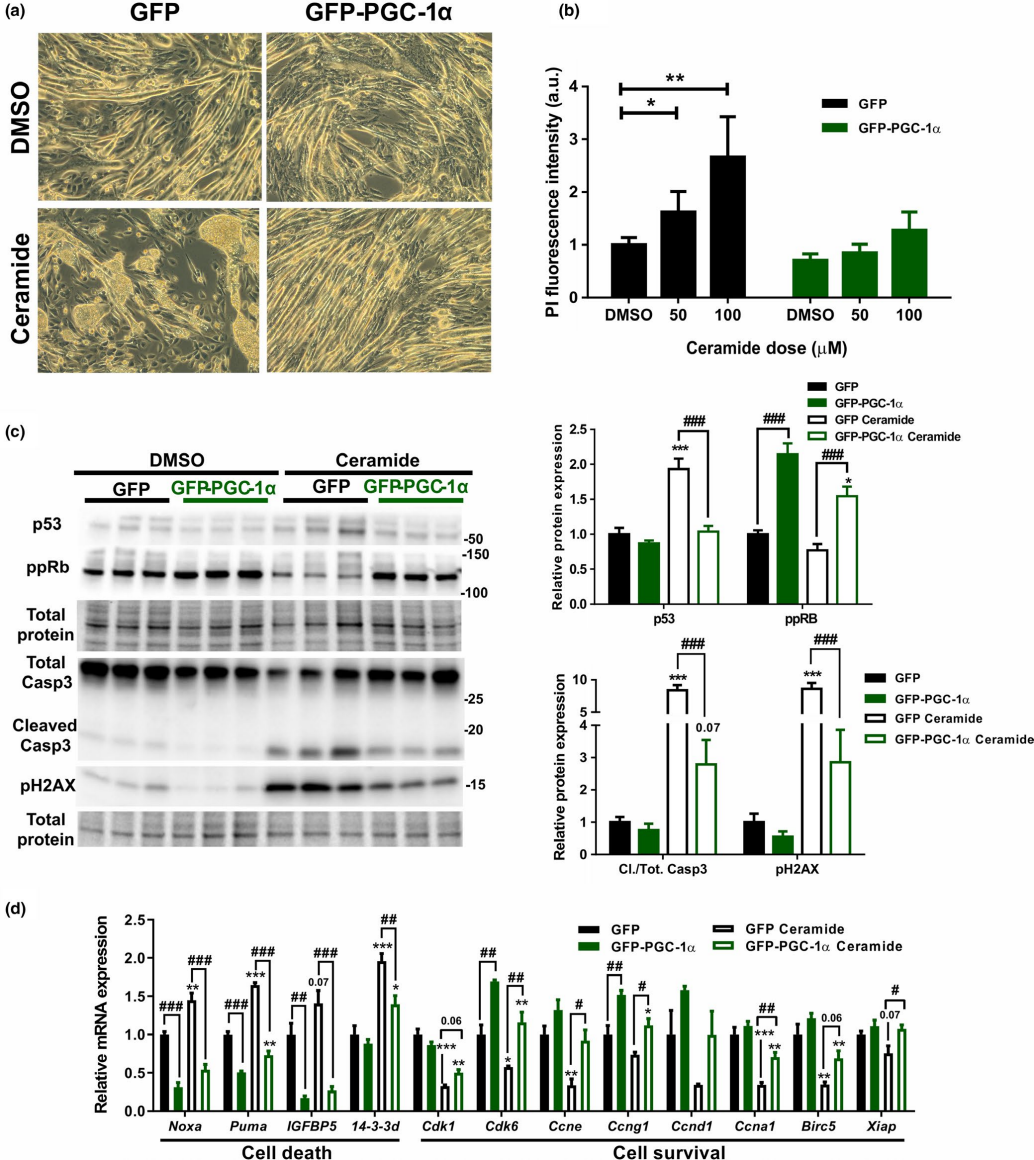
Peroxisome proliferator‐activated receptor γ coactivator 1α (PGC‐1α) protects from ceramide‐induced cell death. (a and b) Representative pictures of myotubes and propidium iodide incorporation in myoblasts with endogenous or increased PGC‐1α levels after ceramide or DMSO treatment. (c) Relative protein levels of P53 and ppRB in myotubes and caspase 3 and pH2AX in myoblasts. (d) Relative myotube mRNA levels of cell death and cell survival markers (*n* = 3 independent experiments with three technical replicates). Values are mean ± *SEM*. *p < 0.05; **p < 0.01; ***p < 0.001 indicate statistically significant differences between cells treated with DMSO and ceramide, ^#^ *p* < 0.05; ^##^ *p* < 0.01; ^###^ *p* < 0.001 indicate statistically significant differences between cells with endogenous and overexpressed PGC‐1α levels

To evaluate the direct influence of PGC‐1α on cell death, we used ceramide, which acts as a second messenger for apoptosis linked to ER stress, mitochondrial impairment, and dysregulation of calcium homeostasis (Jarvis, Grant, & Kolesnick, 1996), hence a number of events that we also observed in muscle aging in vivo. We observed that PGC‐1α overexpression protected C2C12 cells following ceramide exposure as evaluated by microscopy and by a propidium iodide‐based cell death assay (Figure 5a,b). Of note, similar findings have been reported in HeLa cells (Bianchi et al., 2006). Furthermore, PGC‐1α overexpression in muscle cells abolished the induction of p53, reduced the cleavage of caspase 3 proteins, and alleviated the increase in expression of the DNA damage marker phospho‐H2A histone family member X (pH2AX) in response to ceramide treatment (Figure 5c). Inversely, protein levels of the phosphorylated form of the phospho‐prepro‐retinoblastoma‐associated protein (ppRb), which represents an inactive state of this protein involved in triggering apoptosis upon DNA damage (Ianari et al., 2009), were increased by PGC‐1α (Figure 5c). Moreover, PGC‐1α upregulation abrogated ceramide‐dependent changes of gene expression related to cell death and survival (Figure 5d). Next, we assessed whether PGC‐1α protects myocytes from cell death specifically induced by cytosolic calcium dysregulation. Therefore, we treated myocytes with thapsigargin (TPG), a noncompetitive inhibitor of the ATPase sarcoplasmic/endoplasmic reticulum Ca2+ transporting (SERCA), which blocks SR calcium uptake and promotes apoptosis through a dramatic increase in cytosolic calcium and ER stress (Chen, Chiang, Hung, Hung, & Lai, 2000). Similar to its protective role after ceramide treatment, PGC‐1α protected muscle cells from TPG‐induced apoptosis (Figure S5a,b). This protective effect was accompanied by a reduction of TPG‐mediated caspase 3 activation and pH2AX protein elevation (Figure S5c). At the same time, the downregulation of ppRB protein levels by TPG was prevented by PGC‐1α overexpression (Figure S5c). Notably, unlike ceramide, TPG did not affect p53 protein levels, indicating that ceramide and TPG induce apoptosis through at least in part different pathways, both of which are affected by PGC‐1α. Together, these data suggest an essential role for PGC‐1α in the prevention of cell death.

## 3 DISCUSSION

Peroxisome proliferator‐activated receptor γ coactivator 1α is a key regulator of mitochondrial biogenesis, dynamics, and function in young animals. Moreover, the effects of this transcriptional coactivator extend to other organelles and cell compartments, such as the unfolded protein response after exercise (Wu et al., 2011) and SR‐controlled calcium homeostasis (Summermatter et al., 2012). We describe here a novel PGC‐1α‐regulated pathway involving the collective control of the function and interaction between mitochondria and the SR centered on calcium homeostasis. Our findings accordingly provide a mechanistic and functional link between the observed decline in PGC‐1α expression and the ensuing reduction in mitochondrial dynamics and activity in muscle at old age that have been reported previously.

Mitochondrial calcium uptake is important for electron transport chain function and hence ATP production, but also significantly contributes to the modulation of intramyocellular resting calcium levels (Rizzuto et al., 2012). For example, the age‐related decline in mitochondrial calcium buffering capacity leads to a rise in intracellular calcium levels in old muscle (Fernandez‐Sanz et al., 2014; Pietrangelo et al., 2015). The improved calcium buffering capacity of mitochondria from muscles overexpressing PGC‐1α can therefore blunt much of the stress exerted by increased intracellular calcium in this context. Besides direct mitochondrial calcium uptake, improved mitochondrial function and ATP production help to maintain calcium reuptake into the SR via the ATP‐dependent calcium pumps (Allen, Lamb, & Westerblad, 2008). Concurrently, the reduction in poly‐ubiquitinated proteins and the expression of Xbp1 and BIP indicate a PGC‐1α‐dependent alleviation of the SR burden. Complete abrogation of tubular aggregates formation by PGC‐1α further illustrates the muscle protection from ER stress development and escalation to tubular aggregates during aging.

Tubular aggregates are predominantly composed of small, densely packed tubules arising from SR (Chevessier, Marty, Paturneau‐Jouas, Hantai, & Verdiere‐Sahuque, 2004) and are associated with both natural and premature aging in mice (Agbulut et al., 2000). In humans, tubular aggregates have likewise been reported in old muscle (Tomonaga, 1977), but are also prominently observed in several other pathological conditions, including peripheral neuropathies, amyotrophic lateral sclerosis and myotonic dystrophy (Chevessier et al., 2005; Schiaffino, 2012). Moreover, these abnormal structures represent the predominant symptom in tubular aggregate myopathy (Schiaffino, 2012). Due to their calcium loading capacity, tubular aggregates are alternatively thought to be a compensatory mechanism to counteract age‐associated cellular calcium increase, rather than a consequence of dysregulated calcium metabolism. Here, we report that PGC‐1α overexpression fully protects muscle from tubular aggregate formation in old mice, while muscle ablation of the PGC‐1α gene markedly exacerbates tubular aggregate occurrence during aging (Figure 3 and Figure S4) or, as previously reported, upon denervation (Vainshtein, Desjardins, Armani, Sandri, & Hood, 2015). Moreover, a link between impaired mitochondrial calcium handling and tubular aggregate formation was reported in young mitochondrial calcium uptake 1 (MICU1) knockout mice, which recapitulate some of the phenotype of old WT and young and old PGC‐1α knockout muscle, including mitochondrial dysfunction, impaired muscle function and the formation of tubular aggregates (Liu et al., 2016). In parallel to improving calcium homeostasis and downregulation of proteins that accumulate in tubular aggregates such as CSQ1, a central role for PGC‐1α in this context is further substantiated by our SDH staining and electron microscopy data as well as other studies that revealed a strong preference for tubular aggregate formation in glycolytic fibers (Funk et al., 2013). The potent effect of PGC‐1α in driving an oxidative muscle fiber shift (Gill et al., 2018; Handschin, Chin, et al., 2007; Lin et al., 2002) thus likely further contributes to the inhibition of tubular aggregate formation. In light of our data, it would be interesting to study whether increased muscle PGC‐1α levels prevent tubular aggregate formation in mice with tubular aggregate‐associated myopathies.

Mitochondrial dysfunction, ATP depletion, calcium dysregulation, and ER stress, as observed in old WT and mKO‐PGC‐1α mice, are strong promoters of cell death (Danial & Korsmeyer, 2004). The protective effect of muscle PGC‐1α on mitochondrial and SR function could thus directly or indirectly result in the observed reduction of cell death initiation. Similar to our in vivo observations, the induction of cell death in C2C12 cells upon ER and mitochondrial stressors (e.g., as evoked by ceramide) or abnormal cellular calcium elevation (e.g., as triggered by TPG) is inhibited when PGC‐1α is overexpressed. Interestingly, the anti‐apoptotic function of PGC‐1α has previously been suggested in a number of tissues, including the retina (Egger et al., 2012), vascular endothelial cells (Valle, Alvarez‐Barrientos, Arza, Lamas, & Monsalve, 2005), neurons (Luo, Zhu, Jia, Zhang, & Xu, 2009), and skeletal muscle (Adhihetty et al., 2009). Our findings now provide direct evidence of a broader PGC‐1α‐controlled program that links functional mitochondria, SR, and calcium handling to cell death regulation in muscle, which becomes compromised with decreased PGC‐1α expression during aging, and likely leads to higher rates of myofiber apoptosis. Indeed, decreased levels of various indicators in old PGC‐1α overexpressing animals strongly suggest that PGC‐1α protects muscle against age‐related cell death. The effect of PGC‐1α on P53, pH2AX, and ppRb expression that we described are reminiscent of findings in vascular endothelial cells, in which a telomere‐P53‐PGC‐1α axis has been postulated to be involved in regulating apoptosis and senescence (Sahin et al., 2011; Xiong, Patrushev, Forouzandeh, Hilenski, & Alexander, 2015). A similar signaling cascade could accordingly be involved in modulating DNA damage and cellular senescence in old skeletal muscle.

**Figure 6:**
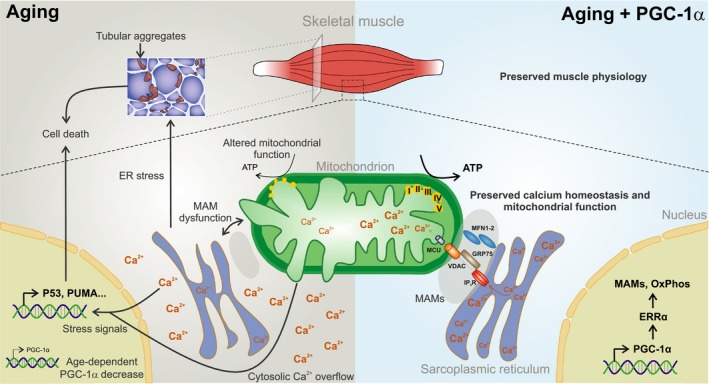
Peroxisome proliferator‐activated receptor γ coactivator 1α (PGC‐1α) regulation of calcium and cell death during skeletal muscle aging. Aging and PGC‐1α downregulation impair mitochondrial respiration, calcium import, and sarcoplasmic reticulum (SR)‐association leading to exacerbated cytosolic calcium increase and SR stress, which ultimately results in tubular aggregate formation. Those cellular dysregulations all contribute to promote muscle apoptosis. PGC‐1α elevation improves mitochondrial function and calcium buffering, preserving calcium homeostasis and preventing tubular aggregate development during aging. Together with the reduction of those cellular insults, PGC‐1α inhibits apoptosis initiation, thereby further protecting muscle from cell death

In summary, our results demonstrate that PGC‐1α, in close relation with ERRα, is central in the coordinated control of SR and mitochondrial calcium homeostasis, thereby preventing SR stress and activation of cell death pathways (Figure 6). Intriguingly, this control is exerted in a context‐specific manner: For example, while PGC‐1α upregulates the unfolded protein response to cope with the acute consequences of exercise (Wu et al., 2011), increased muscle PGC‐1α in old muscle alleviates the aging‐related burden on the SR by lowering the ER stress response. Accordingly, mKO‐PGC‐1α mice in many regards exhibit a premature aging phenotype (present results and ref. Gill et al., 2018). Along the same line, these animals have previously been reported to suffer from exacerbated age‐related glucose intolerance and systemic inflammation (Sczelecki et al., 2014). This is different from cardiac tissue, where a reduction in PGC‐1α gene expression has a limited effect on aging, mainly affecting mitochondrial gene expression (Whitehead, Gill, Brink, & Handschin, 2018). Inversely, PGC‐1α elevation improved both cardiac (Whitehead et al., 2018) and skeletal muscle function in old mTg‐PGC‐1α mice, overall positively affecting health‐adjusted life expectancy (health span), thus the length of time of life in a healthy state, as evidenced by preserved locomotion and exercise capacity (Figure 1f,g), as well as balance and motor coordination (Gill et al., 2018). Our findings are furthermore corroborated by a recent publication reporting a delayed aging process of mTg‐PGC‐1α animals in terms of global gene expression patterns, markers for mitochondrial function and muscle wasting, and morphology of the neuromuscular junction (Garcia et al., 2018). Interestingly, while mTg‐PGC‐1α exhibit such an extension in health span, alteration of muscle PGC‐1α expression did not affect overall lifespan in our study (Figure S6). A modest increase (~5%) in median lifespan has however recently been reported in those mTg‐PGC‐1α mice that reached a minimal age of 800 days, but not when all animals were analyzed (Garcia et al., 2018), indicating a subtle beneficial effect of muscle PGC‐1α on survival in old mice. Collectively, our present findings reveal a novel cascade of cellular processes explaining how PGC‐1α affects muscle health in aging, involving SR‐mitochondrial interaction linked to calcium handling, SR stress, and cell death pathway activation (Figure 6). In light of our present and other recent findings (Garcia et al., 2018; Gill et al., 2018; Sczelecki et al., 2014), PGC‐1α modulation, for example, by exercise or pharmacological interventions, represents an attractive approach to reduce weakness, frailty, and other pathological alterations associated with skeletal muscle aging, along with additional potential benefits on the heart (Whitehead et al., 2018).

## 4 EXPERIMENTAL PROCEDURES

### 4.1 Animals and reagents

Mice with muscle‐specific PGC‐1α deletion (mKO‐PGC‐1α) or overexpression (mTg‐PGC‐1α) were previously described (Handschin, Chin, et al., 2007; Handschin, Choi, et al., 2007; Lin et al., 2002). C57/Bl6 wild‐type (WT) mice were obtained from Janvier. Male mice were studied at 3 and 24 months of age unless otherwise stated. All experiments were performed in accordance with the federal guidelines for animal experimentation and were approved by the Kantonales Veterinäramt of the Kanton Basel‐Stadt.

Primary antibodies were obtained from Cell Signaling, Thermo Scientific, Abcam, Sigma, or Enzo, and secondary antibodies from Dako, Jackson Immunoresearch, or Life Technologies. General reagents were purchased from Sigma‐Aldrich. Detailed methodological information about muscle preparation, physical activity, histology, DNA/RNA extraction and qPCR, protein extraction and Western blots, cell culture experiments, mitochondrial respiration and calcium handling, and statistical analysis can be found in the Supplementary Material.

### 4.2 Transmission electron microscopy

Sample preparation and mitochondrial volume density calculations were performed as previously described (Arnold et al., 2014). Briefly, volume density is defined as the ratio of test points residing within mitochondria and the total amount of test points within the field of view; using the measurement tool in Photoshop, the number of pixels contained within mitochondria was compared to the total number of pixels in each image. Mitochondria were identified and outlined manually. Mitochondrial size was measured as the pixel area contained in each mitochondrion, adjusted to µm^2^ scale corresponding to image magnification. Five images were quantified per block of stained muscle tissue, for a total of five blocks per mouse. Averages of mitochondrial size were taken across all mitochondria measured per mouse.

Further descriptions of methods are included in the Supplementary Material.

## CONFLICT OF INTEREST

M.R. is an employee of Novartis Pharma AG.

## AUTHORS' CONTRIBUTION

J.F.G., J.D., S.M., J.S.P., and C.H contributed to conceptualization of the study. J.F.G., J.D., S.M., J.S.P., M.R., and C.H carried out the formal analysis. J.F.G., G.S., S.F., S.M., M.R., and S.S. involved in investigation. J.F.G, J.D., and C.H wrote the manuscript.
