# Supplementary material for: Peroxisome proliferator‐activated receptor γ coactivator 1α regulates mitochondrial calcium homeostasis, sarcoplasmic reticulum stress, and cell death to mitigate skeletal muscle aging
Source: Aging Cell. 2019 Jul 10;18(5):e12993. doi: 10.1111/acel.12993 (PMC6718523; doi:10.1111/acel.12993)
Supplement: Supplementary file 1 [file ACEL-18-e12993-s001.pdf]

Supplemental Methods

Mouse muscle preparation

Mice were sacrificed by CO_2 inhalation. Muscles were harvested and either snap-frozen in liquid nitrogen for protein and RNA extraction, frozen in cooled-isopentane and embedded in tragacanth for cryosection staining, immediately used for determination of calcium uptake in isolated mitochondria or fixed for electron microcopy.

Treadmill experiment and spontaneous locomotor activity

To assess locomotor performance, mice ran in an open treadmill (Columbus instruments). Mice were acclimatized to the treadmill for 5 min at 8 m/min followed by 5 min at 10 m/min, at an incline of 5° during two consecutive days. After one resting day, an exhaustion test was performed at an inclination of 5° and a starting running speed of 4.8 m/min which was then increased by 1.6 m/min every 3 min until a maximum speed of 29 m/min. Maximal running speed was recorded when exhaustion was reached.

Spontaneous locomotor activity was recorded by counting number of beam breaks in an indirect calorimetric system (CLAMS, Columbus Instruments) in 15 min intervals. Data were analyzed after one day of acclimatization for 2 days.

Histology

Tragacanth-embedded muscles were cut to 8 µm sections with a cryostat (Leica, CM1950). H&E staining was performed as described in the DMD_M.1.2.007 SOP (http://www.treat-nmd.eu/downloads). For SDH staining, sections were incubated in SDH buffer (phosphate buffer 0,035M, Na-succinate 0.1M, teranitro blue trezolium salt 0.1%, phenazin methosulfat 0.1%), rinsed with water, fixed with 4% formalin and rinsed

with water again before being mounted with CC/Mount (Sigma, C9368). For calsequestrin 1 labelling, cryosections were fixed for 20 min with 4% PFA in PBS (Sigma-Aldrich, D8537) at room temperature. Sections were blocked in PBS supplemented with 0.4% Triton X-100 (Sigma-Aldrich, 93426), 3% Goat Serum (Sigma-Aldrich, G9023), 1% BSA (Sigma-Aldrich, A9418). Sections were blocked for 30 min at room temperature and incubated with primary and secondary antibodies for one hour at room temperature. 3 washes of 5 min in PBS were performed before and after antibody incubation. Primary and secondary antibodies were diluted in blocking solution. Calsequestrin 1 (Thermo Scientific, MA3-913) and laminin (Sigma, L9393) antibody dilutions were 1/250 and 1/5000 respectively. AlexaFluor 488 (Life Technology, A-11008) and 647 (A-21242) secondary antibody dilutions were 1/500 and 1/250 respectively. Labelled sections were mounted with ProLong Gold Antifade reagent (Life Technologies, P36931). Images of calsequestrin 1 immunolabellings and H&E stainings were taken at the imaging core facility of the Biozentrum with the FEI MORE microscope using a 20x or 40X magnification lens, keeping the same acquisition settings for all compared samples. Numbers of tubular aggregates were quantified in all fibers of each stained muscles using ImageJ software with original images. Representative images were however adjusted for brightness and contrast.

Genomic DNA/RNA extraction and qPCR

- Genomic DNA

For genomic DNA extraction, crushed gastrocnemius muscles were shaken overnight at 55° in 600 µl lysis buffer (10mM Tris-HCl, 1mM EDTA, 0.1% SDS 5% Proteinase). Lysates were centrifuged at 8000g for 15 min at room temperature and supernatants transferred to fresh tubes. Residual RNA was removed by incubation with RNase A (20mg/ml) for 30 min at 37°C under constant agitation. RNase A was inactivated for

10 min at 95°C and samples were cooled-down to room temperature. One volume of phenol/chloroform/isoamylalcohol 25:24:1 was added and samples were centrifuged at 8000g for 15 min at room temperature. The aqueous phase was transferred to a new tube before addition of one volume of chloroform. The centrifugation step was repeated and the new aqueous phase was transferred again to a new tube where one volume of isopropanol containing 0.3M of sodium acetate was added. Samples were gently mixed and placed at -20°C for 30 min. DNA was recovered by centrifugation at 8000g for 15 min at 4°C. DNA pellets were washed 2 times with 1ml of ice-cold 75% ethanol, dried during few minutes and finally resuspended in ddH2O. An amount of 0.1µg of gDNA was used for qPCR.

- RNA extraction and qPCR

Total RNA was isolated from powdered muscles using lysing matrix tubes (MP Biomedicals 6913-500) and TRI Reagent (Sigma-Aldrich T9424). Total RNA was recovered from cells using the Direct-zol RNA MiniPrep kit (Zymo Research R2050 according to the manufacturer's instructions). After treatment with RNase-free DNase (Invitrogen 18068-015), 1µg of RNA was used for reverse transcription using the SuperScript II reverse transcriptase (Invitrogen 18064-014).

The level of relative genomic DNA or mRNA was quantified by real-time PCR on a Light Cycler 480 system (Roche Diagnostics) using FastStart essential DNA probe master mix (Roche Diagnostics 06402682001). Relative quantification of mRNA for gene expression comparison was performed with the ΔΔCT method using the TATA binding protein (TBP) gene as reference. The quantification of mitochondrial DNA copy number was done using the same method by normalizing the average of COX1 ATP6 and ND1 DNA copy number by the average copy number of the nuclear genes beta

globin and 34B6. Beta globin and TBP levels were similar between genotypes in a given experimental condition. Primer sequences are listed in Supplemental Table S1.

Protein extraction and Western blots

Quadriceps muscles were crushed on dry ice and homogenized in 250µL of ice-cold lysis buffer (50 mM Tris-HCl (pH 7.5), 250 mM sucrose, 0.25% Nonidet P 40 substitute, 1 mM EDTA, 1 mM EGTA, 50 mM NaF, 5 mM Na 0.1% DTT, fresh protease, and phosphatase inhibitor mixture). Samples were then incubated for 30 min at 4 °C under constant agitation at 1,300 rpm, before being centrifuged at 13,000×g for 10 min at 4 °C. The resulting supernatants were transferred to a fresh tube and protein concentrations were measured by Bradford assay (Bio-Rad, 500-0205). Total proteins from C2C12 cells were extracted after an ice-cold PBS wash following the same procedure. Equal amounts of protein were separated on mini-TGX 4-20% stain free pre-cast gel (Biorad, 4568096). Proteins were labelled with trihalo compounds of the stain-free gel by exposing the gel to UV for 1 min. Gel and nitrocellulose membranes were equilibrated for 5 min in transfer buffer and proteins were transferred on the nitrocellulose membrane during 1h under a constant voltage of 100V. Membranes were then blocked 1 h at room temperature with 5% milk or BSA diluted in TBS-T and washed 2 times 5 min with TBS-T. Proteins of interest were then labelled overnight at 4°C with primary antibodies diluted in TBS-T containing 0.02% sodium azide and either 3% milk or 3% BSA. Membranes were washed three times 5 min with TBS-T. Membranes were then incubated 1 h at room temperature with peroxidase-conjugated secondary antibodies diluted in TBS-T containing 3% milk or BSA. Membranes were then washed 3 times for a total of 15 min. Antibody binding was revealed using the enhanced chemiluminescence HRP substrate detection kit SuperSignal™ West Dura Extended Duration Substrate (Thermoscientific, #34076) and imaged using a fusion

FX imager. Proteins of interest were normalized against total protein content determined using the trihalo compounds labeling. Two reference samples were loaded in the different gels for inter-gel normalization. Quantification of proteins was done with fusion FX software. Blocking and antibody solutions, antibody information and dilutions are described for each protein of interest in Supplemental Table S2. All primary antibodies were diluted at 1/1000 except Calsequestrin 1 antibody that was diluted at 1/3000. All secondary antibodies were diluted at 1/10000.

C2C12 growth, differentiation and viral infection

C2C12 cells were cultured DMEM (Sigma-Aldrich D 5796), supplemented with 10% fetal bovine serum (HyClone Laboratories, Inc., Logan, UT), 4.5 mg/ml glucose and 1% penicillin/streptomycin and were incubated at 37 °C with 5% CO2. Myotube differentiation was achieved by incubation of 95% confluent myoblasts in differentiation medium (DMEM with 2% horse serum, 4.5 mg/ml glucose and 1% penicillin/streptomycin) during 4 days. PGC-1α overexpression was performed with adenoviral vectors expressing bicistronic GFP-PGC-1α or GFP alone as control. Infection was initiated in 50% confluent myoblasts or in myotubes after 4 days of differentiation. ERRα knock-down combined with PGC-1α overexpression was performed in C2C12 myoblasts by infecting cells with adenoviral vectors containing specific shRNA sequences against ERRα (shEsrra) or Lacz (shLacz) simultaneously with viruses used to study PGC-1α upregulation.

For cell death experiments, 2 days after infection, muscle cells were treated for 8 hours with 50 µM or 100 µM ceramide (Sigma-Aldrich 01912) or for 24h with 1µM thapsigargin (Sigma-Aldrich T9033). 0.1% DMSO (Sigma-Aldrich 276855) was used as control in both treatments. Pictures of myocytes after treatment were taken using Leica DMI4000B microscope with a 10x magnification. After myoblast exposure to

ceramide or thapsigargin, cell death assays were performed by exchanging treatment medium with fresh medium containing 5 µM of propidium iodide. Cells were incubated for 30 min at 37°C with 5% CO_2 and propidium iodide incorporation reflecting cell death was measured using a tecan infinite M1000 multiplate reader.

Mitochondrial calcium uptake assay

Quadriceps, tibialis anterior (TA) and EDL muscles of both legs from each mouse were pooled together, rinsed 2 times with PBS and minced finely with scissors in 0.5 ml of mitochondrial isotonic buffer (mannitol 225 mM, sucrose 75 mM, MOPS 5 mM, EGTA 0.5 mM, taurine 2 mM, pH 7.25). The resulting muscle homogenates were incubated 3 min in 10ml of mitochondrial isotonic buffer containing nargase at 0.1 mg/ml (Sigma-Aldrich, P8038) before the addition of BSA at 0.2%. Muscle solutions were then transferred in Potter-Elvehjem grinders and homogenized with manual pestles. Homogenates were centrifuged for 6 min at 1200g for 2 times, discarding fat and cellular debris between centrifugations. Obtained supernatants were centrifuged for 10 min at 9000g. Residual fat was discarded and mitochondrial pellets were washed with 15ml of mitochondrial isolation buffer before a second centrifugation at 9000g for 10 min. Final mitochondrial pellets were gently resuspended in a final volume of 150 µl of mitochondrial isotonic buffer. Mitochondrial protein concentrations were determined by Bradford method and 275 µg of mitochondria were centrifuged and gently resuspended in 100 µl of mitochondrial calcium assay buffer (KCl 120 mM, Tris 10 mM, MOPS 5 mM, K_2HPO_4 5 mM, 10 mM Glutamate, 2 mM Malate pH 7.4). 100 µl of calcium buffer containing 5 µM of the fluorescent calcium indicator calcium green 5N (Molecular probes, C3737) was loaded in a 96-well black plate (Nunc) and baseline fluorescence was measured using a Tecan infinite M1000 multiplate reader. When the signal was

stable, mitochondrial preparations were added and calcium green 5N fluorescence was continuously recorded during subsequent addition of calcium.

FDB fiber isolation and calcium measurements

The flexor digitorum brevis (FDB) muscle was dissected manually from 28 months old male mice anaesthetized with isoflurane (4%) and killed by cervical dislocation. The FDB muscle was enzymatically dissociated for 1 hour in Tyrod's buffer (138 mM NaCl, 2 mM CaCl2, 1mM Mg acetate, 4 mM KCl, 5 mM glucose, 10 mM HEPES, pH 7.4) containing 2.2 mg/ml collagenase I (Sigma-Aldrich) in the incubator at 37 °C and 5% CO2. After incubation with collagenase, muscle fibers were manually isolated using fire polished pipette tips and transferred onto matrigel coated 35mm glass bottom dishes (Ibidi GmbH, Martinsried, Germany). The muscle fibers were kept in Dulbecco's Modified Eagle Medium (DMEM) supplemented with 10 % FCS and 1 % penicillin-streptomycin in the incubator at 37°C for 3-4 hours before medium was exchanged.

For the assessment of mitochondrial calcium uptake, FDB fibers were stained with Rhod-2 AM (ThermoFischer Scientific, Waltham, US) dissolved in Tyrod's buffer for 20 min at room temperature. Calcium flux into mitochondria was measured after one single electrical stimulation event, thereby preventing energetic limitation of the muscle fiber without pharmacological modulation of SERCA activity. The final concentration of the dye in buffer was 1µM. During the calcium measurements N-benzyl-p-toluene sulphonamide (BTS, Sigma-Aldrich) was added to the buffer at a concentration of 10 µM to prevent muscle fiber contractions, without affecting calcium release.

Calcium imaging was carried out on an inverted Olympus FV3000-IX83 microscope equipped with a 20x UPSAPO lens. A uni-directional line scan mode with a temporal resolution of 0.488ms/line (2000 lines in total) was used to measure the temporal

profile of the fluorescence signal. All experiments were carried out at room temperature, which was kept constant at 20 °C. Calcium release from the sarcoplasmic reticulum of FDB fibers was induced by a 1ms, 9V single electrical pulses using linear platinum electrodes that were placed at the edges of the glass bottom dish in about 1cm distance from each other. Typically, 50 fibers from one dish could be analyzed within a 20min time frame.

For image analysis we used Icy (open source software created by the Quantitative Image Analysis Unit at Institut Pasteur, Paris, France) in combination with the CalciumFluxAnalysis plugin to quantify the fluorescent intensity profiles in terms of amplitude (ΔF/F) and kinetics. ΔF/F is the relative measure of the amount of calcium flux into the mitochondria during a transient event such as a single pulse electrical stimulation. The statistical analysis was carried out in Systat (Systat Software, Inc.).

Statistical analysis

Data were analyzed with two-way ANOVA (GraphPad Prism software). Sidak post-tests were used for multiple comparison analysis following two-way ANOVA. All data are plotted as mean ± S.E.M. For calcium measurements in FDB fibers, the data points of one group are not independent as they are derived in part from the same animal (random factor). Therefore, statistical tests were based on a mixed model analysis (SYSTAT 13) for those experiments and the animal group was used as the fixed factor in each statistical analysis.

Supplemental Tables

Table S1. Primer Sequences for RT-PCR Analysis

Gene name	forward sequence	reverse sequence
**βglobin**	GAAGCGATTCTAGGGAGCAG	GGAGCAGCGATTCTGAGTAGA
**14-3-3d**	CGAAGACTAGGAGGAGGCAG	CTCTCCATGACTGCGAGGAT
**36B4**	ACTGGTCTAGGACCCGAGAAG	TCAATGGTGCCTCTGGAGATT
**ATP6**	AGTATGAGCTGGAGCCGTAATTACA	TGGAAGGAAGTGGGCAAGTG
**BIRC5**	GAGGCTGGCTTCATCCACTG	CTTTTTGCTTGTTGTTGGTCTCC
**BNIP3**	AAATTAAAGGGTGCGTGCGG	CAAAGTGGGGTTCGTGGGTA
**CDK1**	AGGTACTTACGGTGTGGTGTAT	CTCGCTTTCAAGTCTGATCTTCT
**CDK6**	GGCGTACCCACAGAAACCATA	AGGTAAGGGCCATCTGAAAACT
**COX1**	TGCTAGCCGCAGGCATTACT	GCGGGATCAAAGAAAGTTGTG
**CSQ1**	ACTCAGAGAAGGATGCAGCT	CTCTACAGGGTCTTCTAGGA
**CSQ2**	AGCTTGTGGAGTTTGTGAAG	GGATTGTCAGTGTTGTCCC
**CyclinA**	GCCTTCACTCATTGCTGGAG	TGTTGTGCCAATGACTCAGG
**CyclinD**	GTTCATTTCCAACCCACCCTC	AGAAAGTGCGTTGTGCGGTAG
**CyclinE**	CCCTCTGACCATTGTGTCCT	TCGCACCACTGATAACCTGA
**CyclinG1**	GTTCACGACACCTTGCCATT	AGAAGGTCAAATCTCGGCCA
**DRP1**	GCGCTGATCCCGCGTCAT	CCGCACCCACTGTGTTGA
**ERRα**	CGGTGTGGCATCCTGTGA	CTCCCCTGGATGGTCCTCTT
**FIS1**	GCCCCTGCTACTGGACCAT	CCCTGAAAGCCTCACACTAAGG
**GRP75**	TGACCAAAGACAACATGGCG	TAGCTTTCTGACACGGAGCA
**I3PR1**	GCCTTGCTAGAGAAGAACGC	CATTGCAGCCTGGGTTATCC
**IGFBP5**	ATACAACCCAGAACGCCAGCT	ACCTGGGCTATGCACTTGATG
**LETM1**	CTCTGAGGCTGTGAAGGACA	CACCCTTCAGACCTTCCAGT
**MCAD**	AACACTTACTATGCCTCGATTGCA	CCATAGCCTCCGAAAATCTGAA
**MCU**	AAAGGAGCCAAAAAGTCACG	AACGGCGTGAGTTACAAACA
**MFN1**	CTGCTTCCTGAGTGTCGAGG	GCATGGGCCAGCTGATTAAC
**MFN2**	GGTCAGGGGTATCAGCGAAG	TTGTCCCAGAGCATGGCATT

**MICU1**	ACACCCTCAAGTCTGGCTTAT	TTCCCATCTTTGAAGTGCTTCTT
**NOXA**	ACTGTGGTTCTGGCGCAGAT	TGAGCACACTCGTCCTTCAAGT
**ND1**	TCTGCCAGCCTGACCCATA	GGGCCCGGTTTGTTTCTG
**NRF1**	TCCCCCGAGGACACTTCTT	ATCAGCTGCCGTGGAGTTG
**OPA1**	CTTGCCAGTTTAGCTCCCGA	CAATTTGGGACCTGCAGTGAA
**OPA2**	CCCAGCTCAGAAGACCTTGC	CCAGGTGAACCTGCAGTGAA
**p21**	GACACCACTGGAGGGTGACT	GGATTAGGGCTTCCTCTTGG
**P53**	GGGACAGCCAACTCTGTTATG	CTGTCTTCCAGATACTCGGGA
**PGC-1α**	AGCCGTGACCACTGACAACGAG	GCTGCATGGTTCTGAGTGCTAAG
**PGC-1β**	CCATGCTGTTGATGTTCCAC	GACGACTGACAGCACTTGGA
**PUMA**	ATGGCGGACGACCTCAAC	AGTCCCATGAAGAGATTGTACATGAC
**SDH**	GCTGGTGTGGATGTCACTAAGG	CCCACCCATGTTGTAATGCA
**SERCA1**	AGCCAGTGATGGAGAACTCG	CACCACCAACCAGATGTCAG
**SERCA2**	GAGAACGCTCACACAAAGACC	CAATTCGTTGGAGCCCCAT
**TBP**	ATATAATCCCAAGCGATTTGC	GTCCGTGGCTCTCTTATTCTC
**TFAM**	GGTCGCATCCCCTCGTCTA	GGATAGCTACCCATGCTGGAAA
**XBP1**	TGGCCGGGTCTGCTGAGTCCG	GTCCATGGGAAGATGTTCTGG
**XIAP**	GCTTGGCGCGAAAAGGTGG	TTGCACGGTGTCTCCTTCAC

Table S2. Immunoblotting conditions

		Primary antibody			Secondary antibody			
**Protein of interest**	**Blocking solution**	**Prod. #**	**Company**	**Solution**	**Antibody name**	**Product #**	**Company**	**Solution**
**Calsequestrin 1**	milk	MA3-913	Thermoscientific	BSA	Gt anti-ms	115-035-146	Jackson	milk
**pH2AX**	BSA	ab11174	Abcam	BSA	Gt anti-ms	115-035-146	Jackson	BSA
**pprB**	milk	3590	Cell signaling	BSA	Sw anti-rb	P0399	Dako	milk
**Caspase 3**	milk	9662	Cell signaling	BSA	Sw anti-rb	P0399	Dako	milk
**P53**	milk	2524	Cell signaling	Milk	Gt anti-ms	115-035-146	Jackson	milk
**VDAC**	BSA	4661	Cell signaling	BSA	Sw anti-rb	P0399	Dako	BSA
**OXPHOS protein**	BSA	ab110413	Abcam	BSA	Rb anti-ms	P0260	Dako	BSA
**poly-ubiquitination**	BSA	BML-PW8810-0500	Enzo	BSA	Rb anti-ms	P0260	Dako	BSA
**BIP**	BSA	ab21685	Abcam	BSA	Sw anti-rb	P0399	Dako	BSA

**Caspase 12**	BSA	2202	Cell signaling	BSA	Sw anti-rb	P0399	Dako	BSA

Supplemental figures

Fig. S1. PGC-1α prevents mitochondrial dysregulation during aging and muscle dysfunction. (a) Relative PGC-1α mRNA levels in different muscles (n=6). **(b)** Relative protein levels of OXPHOS genes (n=6). **(c and d)** Maximal running speed and locomotor activity raw data of corresponding Fig. 1g and f (n=8-12).Values are mean ± SEM. *P < 0.05; **P < 0.01; ***; P < 0.001 indicate statistically significant differences between young and old animals of the same genotype, # p<0.05; ## p<0.01; ### p<0.001; indicate statistically significant differences between genotypes of age-matched animals.

Fig. S2. PGC-1α improves mitochondrial biogenesis and gene expression of mitochondrial metabolism. (a) Electron microscopic pictures representative of mitochondrial mass and quantification, scale bars represent 1 μm (n=6). **(b)** Relative mitochondrial DNA copy number (n=6). **(c)** Mitochondrial size (n=6) **(d)** Relative muscle mRNA levels of mitochondrial biogenesis genes (n=6). **(e)** Full Graph of data represented in Fig.2d. Values are mean ± SEM. *P < 0.05; **P < 0.01; ***; P < 0.001 indicate statistically significant differences between young and old animals of the same genotype, # p<0.05; ## p<0.01; ### p<0.001; #### p<0.0001 indicate statistically significant differences between genotypes of age-matched animals.

Fig. S3. PGC-1α lowers ER stress in muscle during aging and in C2C12 cells. (a) Relative muscle protein poly-ubiquitination during aging (n=6). **(b and c)** Relative myotube xbp1 gene expression and protein poly-ubiquitination (n=3 independent experiments with 3 technical replicates). Values are mean ± SEM. *P < 0.05; **P < 0.01; ***; P < 0.001 indicate statistically significant differences between young and old animals of the same genotype, # p<0.05; ## p<0.01; ### p<0.001 indicate statistically

significant differences between genotypes of age-matched animals or between cells with endogenous and overexpressed PGC-1α levels.

Fig. S4. Tubular aggregates form preferentially in fast fibers and PGC-1α prevents the formation of abnormal structures. (a and b) Representative electron microscopic pictures of gastrocnemius and soleus muscles of young and old animals. **(c)** Representative pictures of SDH staining in old tibialis anterior muscles, scale bar represents 50 μm. **(d)** Representative electronic microscopic pictures of abnormal structures in gastrocnemius muscles of old animals.

Fig. S5. PGC-1α inhibits TPG-induced cell death. (a and b) Representative pictures and propidium iodide incorporation in myoblasts with endogenous or increased PGC-1α levels after TPG or DMSO treatment. **(c)** Relative myoblast protein levels of cell death and cell survival markers after TPG or DMSO treatment. (n=3 independent experiments with 3-4 technical replicates). Values are mean ± SEM. *P < 0.05; **P < 0.01; ***; P < 0.001 indicate statistically significant differences between cells treated with DMSO and TPG, # p<0.05; ## p<0.01; ### p<0.001 indicate statistically significant differences between cells with endogenous and overexpressed PGC-1α levels.

Fig. S6. Overall lifespan is not affected by muscle PGC-1α. Survival curve for WT, mKO-PGC-1α and mTg-PGC-1α animals.

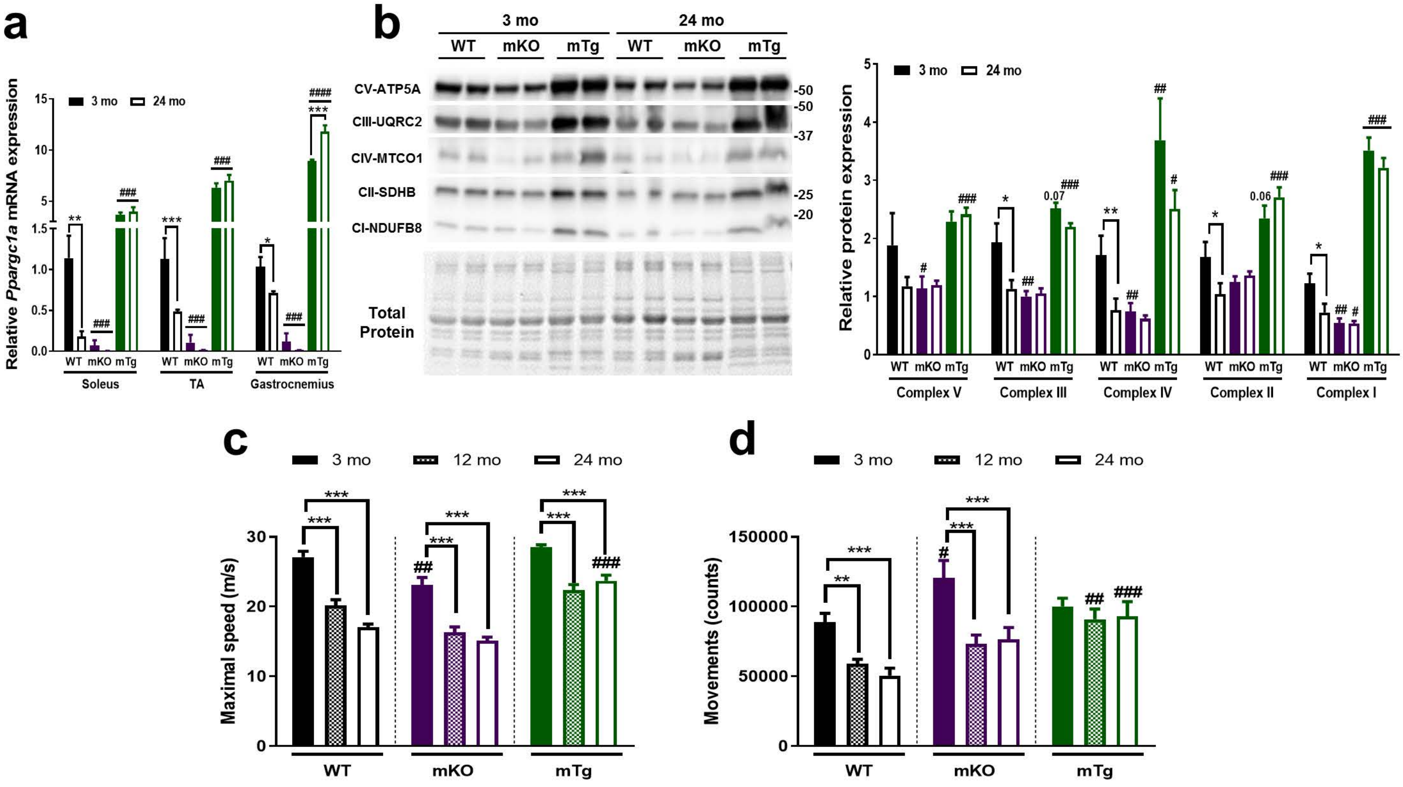
a
Relative Ppargc1a mRNA expression
3 mo
24 mo
WT mKO mTg
Soleus
TA
Gastrocnemius
b
3 mo
24 mo
WT
mKO
mTg
CV-ATP5A
CIII-UQRC2
CIV-MTCO1
CII-SDHB
CI-NDUFB8
Total Protein
-50
-50
-37
-25
-20
Relative protein expression
Complex V
Complex III
Complex IV
Complex II
Complex I
0.07
0.06
c
3 mo
12 mo
24 mo
Maximal speed (m/s)
WT
mKO
mTg
d
Movements (counts)

Suppl. Fig. S1

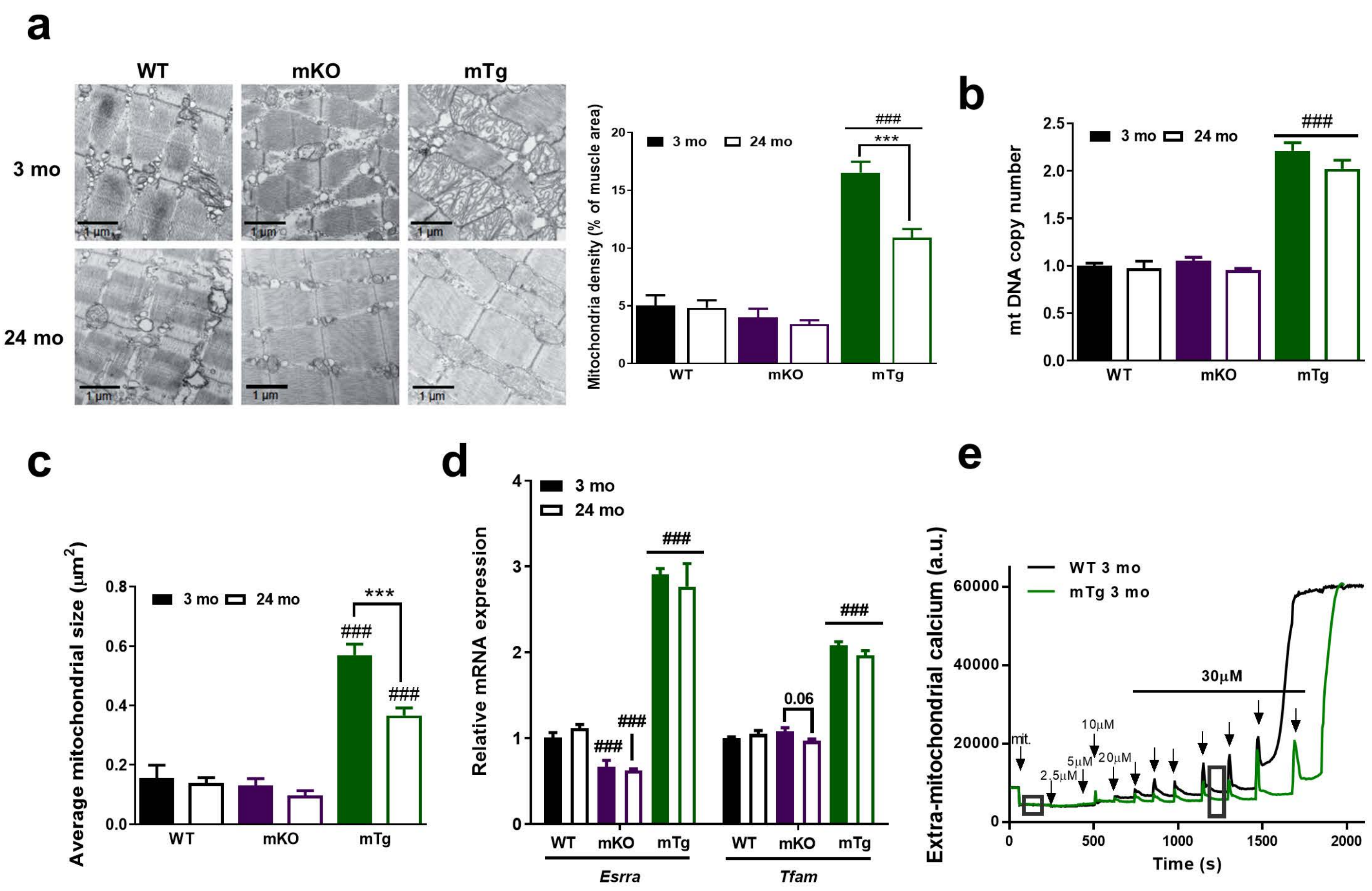
a
WT
mKO
mTg
3 mo
24 mo
1 µm
Mitochondria density (% of muscle area)
3 mo
24 mo
###
***
WT
mKO
mTg
b
mt DNA copy number
3 mo
24 mo
###
WT
mKO
mTg
c
Average mitochondrial size (µm2)
3 mo
24 mo
***
###
###
WT
mKO
mTg
d
Relative mRNA expression
3 mo
24 mo
###
###
###
###
0.06
WT
mKO
mTg
WT
mKO
mTg
Esrra
Tfam
e
Extra-mitochondrial calcium (a.u.)
WT 3 mo
mTg 3 mo
30µM
mit.
2.5µM
5µM
10µM
20µM
Time (s)

Suppl. Fig. S2

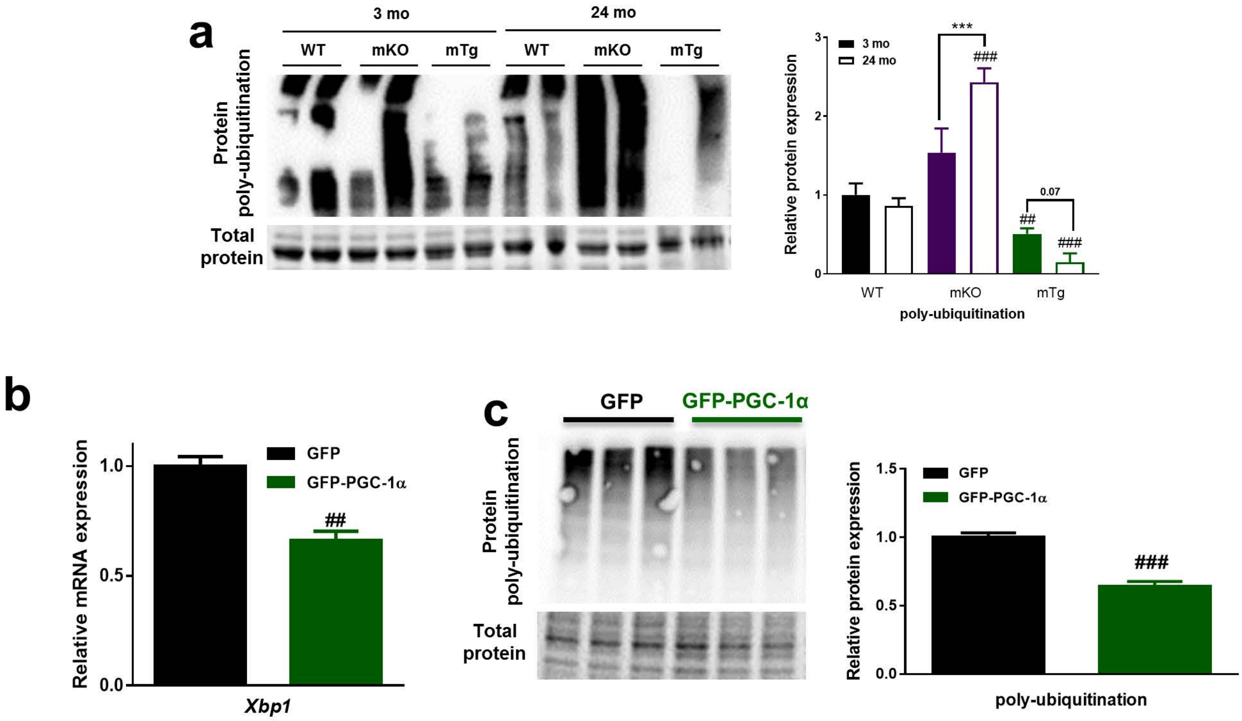

a
3 mo
24 mo
WT
mKO
mTg
WT
mKO
mTg
Protein poly-ubiquitination
Total protein
Relative protein expression
3 mo
24 mo
***
###
0.07
##
###
WT
mKO
mTg
poly-ubiquitination
b
Relative mRNA expression
GFP
GFP-PGC-1α
##
Xbp1
c
GFP
GFP-PGC-1α
Protein poly-ubiquitination
Total protein
Relative protein expression
GFP
GFP-PGC-1α
###
poly-ubiquitination

Suppl. Fig. S3

Suppl. Fig. S4

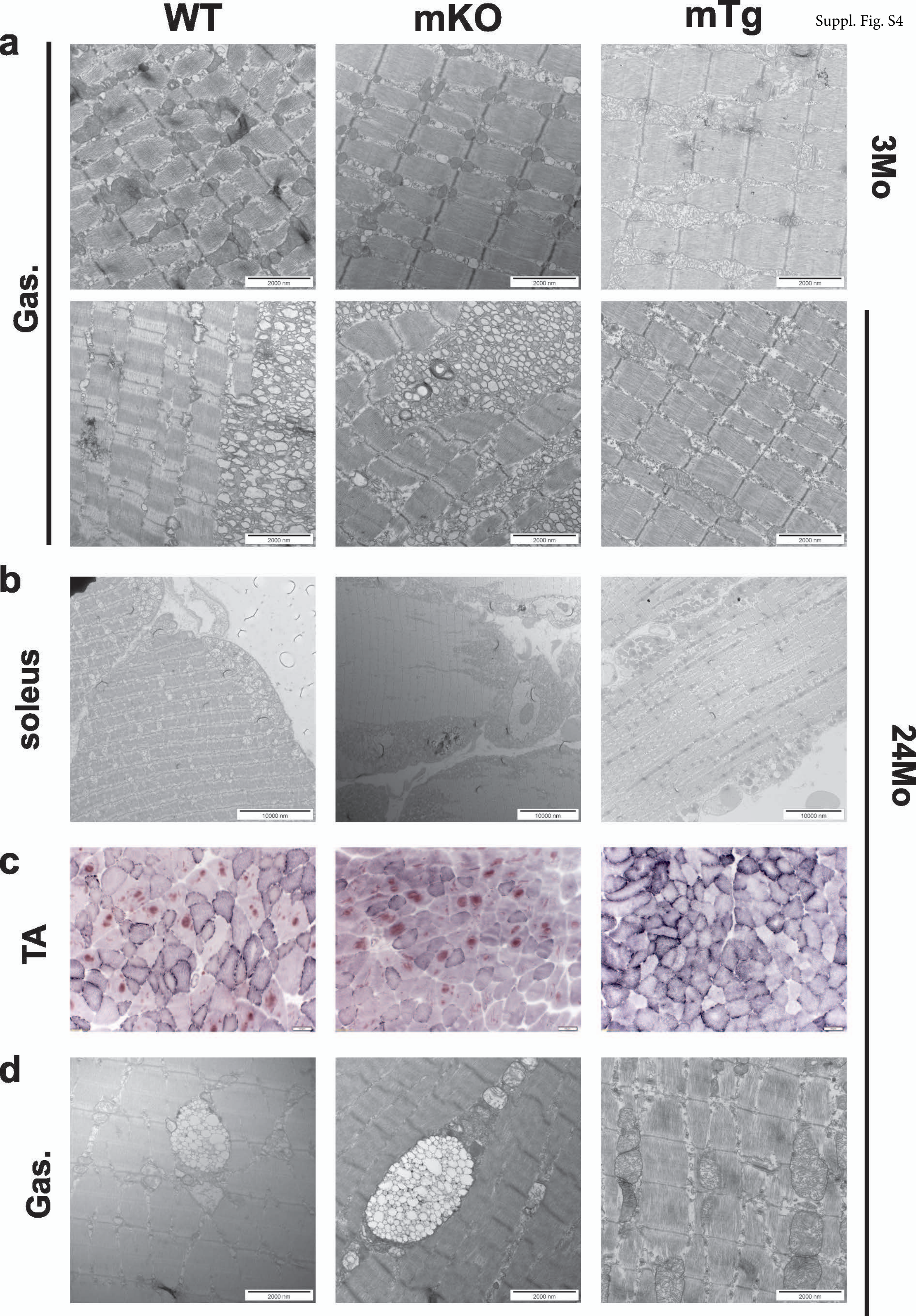

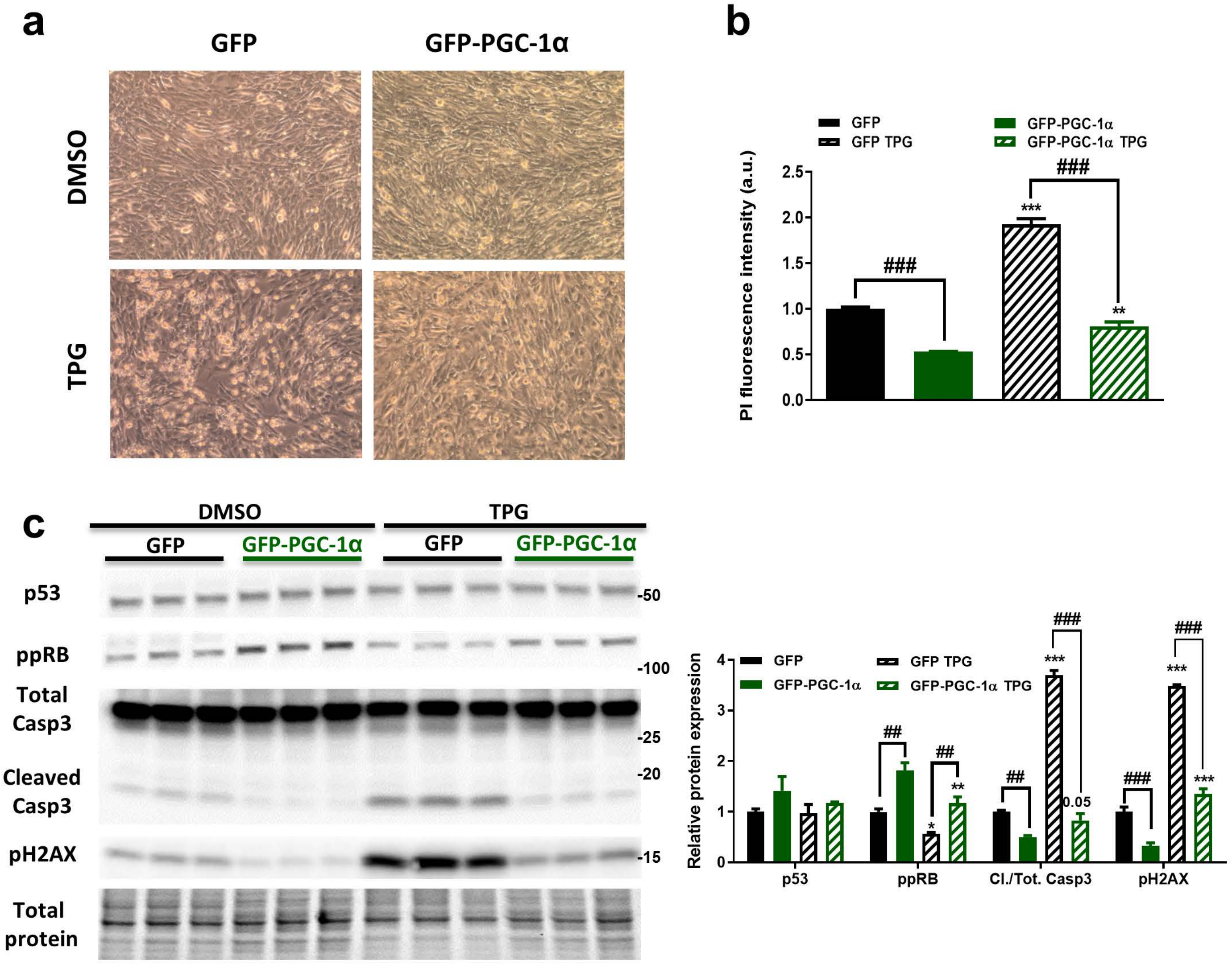
a
GFP
GFP-PGC-1α
DMSO
TPG
b
GFP
GFP TPG
GFP-PGC-1α
GFP-PGC-1α TPG
PI fluorescence intensity (a.u.)
###
***
**
c
DMSO
TPG
GFP
GFP-PGC-1α
p53
ppRB
Total Casp3
Cleaved Casp3
pH2AX
Total protein
-50
-100
-25
-20
-15
Relative protein expression
##
###
*
**
***
0.05
p53
ppRB
Cl./Tot. Casp3
pH2AX

Suppl. Fig. S5

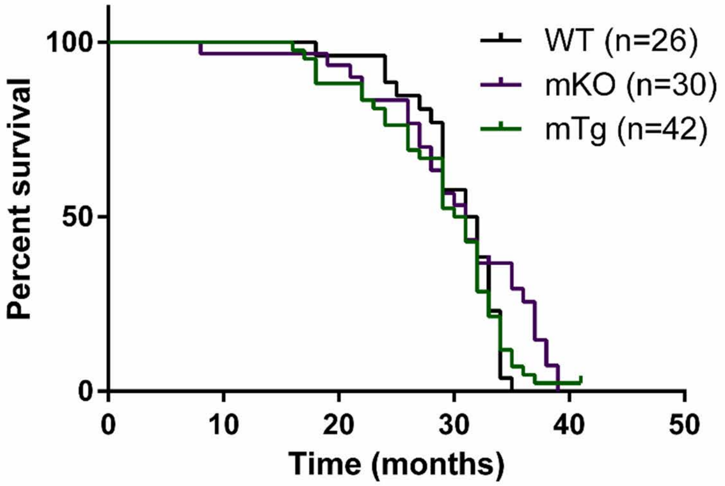

WT (n=26)
mKO (n=30)
mTg (n=42)
Percent survival
100
50
0
0
10
20
30
40
50
Time (months)

Suppl. Fig. S6
